# Analectic

**Published:** 1838

**Authors:** 


### MISCELLANEOUS INTELLIGENCE.

### ANALECTIC, ANALYTICAL, AND ORIGINAL.

### 1.—ANALECTIC.

### AD SYLVAS NUNCIUS.

1. *Observations on the Puriform Opthalmia of the New-Born.* By M. D Carron du Villards.—In this paper I shall consider the ophthalmia of the new-born, more generally known under the name of purulent ophthalmia of children; a name which it has preserved thro' routine and custom, although the illustrious Saunders said, thirty years ago, that the designation of *puriform* should be given to it, on account of the nature of the disease and of the fluid secreted. This ophthalmia, nosologically speaking, ought to be considered as a catarrhal conjunctivitis, which is confined to the mucous membrane until it acquires a certain degree of intensity; then the parts which bear the nearest relation with the conjunctiva become in their turn diseased, a circumstance which gives to this affection all the importance we have pointed out.

In fact, when we devote ourselves for several years to the study of opthalmology, we are astonished at the frequency of this disease, and we are still more frightened to see the number of children who lose their sight in consequence of it.

In a great number of foundling hospitals, and especially in that of Paris, it exists epidemically; and if a great number of children did not sink under the consequences of it, there would be a much greater number of "born blind;" an expression generally admitted, but perfectly false, since the great number of children denominated *blind from birth*, do not come so till after the first months of existence. For a length of time, purulent opthalmia was attributed to syphilis; the assertion with respect to the generality is quite erroneous, since not only authors, but my own experience also, correspond in the conviction that this affection is very rare in children born of infected mothers, whilst it is very frequent in those whose mothers were perfectly healthy. Nay more, I

have observed in a great number of children born of syphilitic mothers, a multitude of venereal phenomena of the skin, on the scalp, on the genitals, and seldom in the eyes; in fact, the discharge must be very copious, in order that the fœtus, passing through the vaginal canal, through the midst of the secretions produced by the accouchement, and surrounded as it is by the amniotic deposits, be infected, since it does not open its eyes till after its passage.

Very often, also, this disease appears one or two months after birth, at a time when the child enjoys good health, or the mother has not even a puerperal discharge. We are very far, however, from denying contagion, since we have seen opthalmia gonorrhœal in the beginning, and then terminating by very decided syphilitic phenomena. As to the possibility of transmission from one individual to the other, it may take place from the child to the mother or to other children. In young animals it follows the same progress; and, in the theses of MM. Chessaignac and Michel, we find very remarkable cases of inoculation of the purulent discharge from eye to eye.

We ought to consider the very peculiar position in which the new-born child is found; scarcely has it cleared the "vaginal *tunnel*," in which it was shut up, at a temperature of 36 deg. and more, of Rheaumur, than it is on a sudden exposed to a decrease of temperature, which is often at least half lower.

This is so true that in a great many circumstances the disease is complicated with a more or less extensive induration of the cellular tissue, coryza, and otorrhœa, to which may be attributed most of the cases of deaf-dumbness. We may invoke also the transgression of hygienic principles, exposure of children to currents of air, or to cold washings. In general, in England, accoucheurs, midwives, and the majority of people out of the profession of medicine, consider this disease as the effect of a change of temperature, known under the name of *a cold*. This opinion is also mine, and in consulting my notes, as well as my recollection, I remember very well that not only at the foundling hospitals of Pavia, Milan, and Paris, but also in private practice, ophthalmia of the new-born is much more common when there are numerous and rapid atmospheric variations. The reason will now be understood why this disease is much more frequent with children of the indigent class, where so many things, even indispensable to life, and still more to the welfare of the new-born individual, are wanting. The irritation of a vivid light plays also a prominent part in the production of purulent ophthalmia. Hence we recommend to persons required to nurse or receive new-born infants, not to expose them to a strong light, nor to lose too much time before they bestow on them hygienic cares. We cannot recommend them too strongly not to wash the eyes with cold water, but to employ tepid water beaten with the yolk of eggs, a mixture admirably adapted for disengaging the face and eyes from the amniotic deposite with which they are often covered.

The child must be clothed sufficiently warm to accustom it gradually to the changes of temperature; and if the infant has remained a long time in the passage, if the face be injected, we must not fail to draw some spoonfuls of blood from the umbilical cord; for Professor Ammon, of Dresden, in researches which are replete with interest, has proved that in a great number of children ophthalmia of the new-born is due to forced injection of the membranes of the eye, accompanied with an insensible sanguineous exhalation, which colors red not only the vitreous humour, but also the crystalline.*

In a great number of circumstances the disease commences by a coryza scarcely sensible, and is betrayed only by slight sneezings. This disease remains stationary for some days, then the eye becomes watery, the eyelids swell, and acquire a rosy tint. At this period, if we examine carefully the small internal fossa of the commissure of the eyelids, commonly known under the name of *larmies*, we perceive in this little anfractuosity a small fragment of dried slightly glutinous mucosity, resembling concrete honey. Gradually this secretion augments, appears in different points of the palpebral commissure, implicates the eyelashes, and glues them together. Then the child begins to experience difficulty in opening the eyelids; they become immovable, and consequently do not allow more of the newly secreted puriform fluid to be thrown out.

When we have seen a certain number of ophthalmiæ in the new-born, we acquire the habit of recognising them from their commencement, before even those who are associated with or nurse the child may have suspected them. It is nearly fifteen years since I saw M. Baron, Physician of the Foundlings, distinguish a puriform ophthalmia, by only observing a red injection across the outer surface of the eyelids; on the other hand the small mucous concretion is a symptom which has never failed me. In proportion as the discharge accumulates between the eyelids, the latter swell and form a kind of pouch, in such a manner, that upon separating them, a milky liquid is seen to ooze instantaneously, which becomes thicker and thicker as the inflammation becomes more intense. Often, at this period, small active hemorrhages take place, which are owing either to interstitial exudations, or to slight venous or arterial ruptures. Now, as the eyelids in the new-born are excessively lax and permeable to liquids, and as the conjunctiva only slightly adheres to the globe of the eye, it is easily invaded by the blood. Then those chemoses are formed every where, accompanied by villosities, which not only produce partial strangulations, but also keep the puriform matter in contact with the cornea, which is then, as is known, soft and thin, and slightly resisting. We ought not to be surprised to see it become dull, assume a grayish tint, become puffed up, ulcerate, split, and in a short time burst, and give rise to all the unpleasant symptoms which accompany those different lesions of the cornea, of which I shall abstain from speaking here.

From the period at which the disease has acquired a certain degree of intensity, the patient is much disturbed by light, and obstinately closes his eyes while he is exposed to it, and not until he is in a dark place does he endeavor to open them. It is truly astonishing that M. Sichel denies this kind of dread of light *(photophobie);* which is ad-

---

*Ammon, Journal d'Ophthalmologie, ii. 23. On the Red Coloration of the Membranes of the Eye in the New-Born, and its influence on the Production of Ophthalmia in the New-Born.

mitted, however, by all those who found their opinion on well-observed facts and not on preconceived ideas. Billard, who had examined with the most scrupulous attention a great number of children affected with puriform ophthalmia, suggested the examination of their eyes during sleep, which may be done with the same facility.*

When we recognise a purulent opthalmia at its commencement, nothing is easier than to limit its action. Some well-understood hygienic cares, lotions of a glassful of cold water acidulated with two or three drops of lemon juice, are enough to afford a sufficient astringency and disperse all traces of purulent discharge. But we should be very careful in applying poultices of crumb of bread, pommes de reinette, white cheese, &c., the results of which are immediately to produce œdema of the eyelids and augment the secretion of mucus.

We should also forbid the nurses to drop their milk into the eye, for this liquid mixed with mucus not only forms a magma which prevents more and more the movements of the eyelids, but also ferments on the spot and becomes a new cause of irritation. For a great number of years I have very promptly put a stop to the inflammation, and mucus secretion, by injecting between the eyelids through a small ivory syringe, some spoonfuls of the following collyrium:—Aqueous infusion of red roses, four ounces; soot, prepared according to my process, eight grains; lemon juice, four drops. I obtained a very rapid cure on the grand-daughter of a *chef de division* of the minister of finances, affected with gonorrhœal ophthalmia fifteen days after her birth, and which was sent to me by a skilful accoucheur, M. Baudelocque, the nephew. At several times during the day the eyelids must be raised to cleanse away the puriform matter. When the disease acquires a certain degree of intensity, we need not be afraid to apply leeches to the temples, and permit the discharge of much blood. Saunders had great confidence in this means, which often succeed with him.

I cannot too strongly blame the conduct of those who, from the example of M. Lawrence, place leeches upon the eyelids themselves.—The motives for my censure repose on the following fact. The slightest traumatic cause is sufficient to induce an œdema of the eyelids in the new-born. The biting of a leech adds to the œdema an ecchymosis and a local puffiness, which does not allow of the eyelid being raised so as to cleanse away the pus. On the other hand, the leech crosses from one part of the eyelid to the other, and wounds the eye; this I have seen frequently happen.

When the symptoms are urgent, we must, in order to arrest the mucous secretion, inject between the eyelids Bates's solution, which possesses very styptic qualities. I have seen this medication succeed wonderfully.

At the same time, we conjoin with this treatment purgative agents, such as the compound syrup of chicory, or rather of peach flowers.

When the disease is decreasing, we may produce a slight rubefaction behind the ears by means of the application of the *pommade* de Lausanne. If by this means we succeed in preventing perforation of the cornea, alarm must not be felt for the transparence of the cornea;

*Billard and Lawrence.---Treatise on Diseases of the Eyes.

this gradually returns of itself, and it has fallen to my lot to see cured, in five or six months, children whom I had considered as entirely deprived of light on account of loss of that transparence.

I have very frequently made use of the solution of nitrate of silver, but with much less advantages than soot, or Bates's solution. When ulcerations, perforations, staphylomas, &c., appear, they must be treated by the usual method. The employment of codfish oil is appropriate for resolving the interlamellar effusions; but to obtain advantage from this medication, all inflammatory symptoms must be watched. I terminate this article in the hope of inducing all practitioners to disperse at their commencement accidents which may bring into jeopardy the eyes of the new-born. I shall account myself extremely happy when I learn that my hope has been realized. It is always with this view that I write.—*Bulletin Gen. de Therapeutique.*—*Dunglison's Intel.*

—

2. *On Inversion of the Uterus.* By Thos. Bradford, Surgeon to the Manchester Lying-in Hospital.

*Causes of Inversion.*—This accident has been attributed to causes purely mechanical, the uterus being unresisting, and passively obedient to their influence. The practice of pulling too early and violently at the funis, after the expulsion of the child, before the uterus has contracted, so as to detach and expel the placenta, has been generally considered as the cause of inversion; but we know that the accident happens before any force has been applied to the funis. * * * * It has occurred when the patient has been delivered of a dead child, the funis so putrid as to break with a very slight effort. It has been found before the cord was separated, and the child given to the nurse. In the practice of Ruysch, this circumstance took place after he had extracted a dead child, &c. These circumstances show that there is a power inherent in the uterus to become inverted. The pulling of the funis is so common a practice amongst our midwives and done without the least consideration of the condition of the uterus, that, if it was so frequent a cause as is usually stated, inversion, instead of being one of the most rare, would be the most common accident in midwifery. Some writers have thought that a short funis is a frequent cause of inversion; whilst others think, in order to act, it must be inserted in the centre of the placenta, and that this mass must be attached to the fundus uteri. It is evident, if brevity of the cord is capable of producing so serious an accident, these peculiarities will greatly add to its influence. But, amongst the published cases of inversion, there is, so far as the writer knows, but one where this shortness existed. It often occurs without diminished length in the cord; whilst, on the contrary, children are frequently born where it is very short, and yet no such event happens. The funis has been ruptured, and the placenta disrupted, and yet the uterus was not inverted.

In order that the causes which have been now alluded to could operate effectually to produce inversion, there must be such condition of the uterus present that it becomes tacitly obedient to their influence. Most systematic writers, as also others, have supposed such to be the case. They have said that the uterus, previous to inversion, is in a state of extreme relaxation, exhaustion, or collapse, and that it offers no resistance to any force applied by the funis. These opinions are at variance with that of the writer.

It appears to the writer that the uterine pain, diminution of bulk, firm resisting feel, sudden formation, and rapid protrusion, warrant him in the deduction that the *fundus* and *body* of the uterus, so far from being in a state of *collapse* or *relaxation*, are really in a state of *unnatural excitement* and *action*. But this is not the case with the os uteri; on the contrary, it is soft and yielding, as we find it offers no resistance to the coming down of the tumour, whose protrusion is forcible and rapid. If these statements be true, it is evident that the fundus and os uteri are in directly opposite conditions: the former is in a state of violent contraction, the latter in a state of relaxation; and that this relative difference in these two parts of the organ is indispensably necessary to exist where inversion occurs.

From what has been stated, it may be concluded that quick labor, whether natural or artificial,—a disturbance of this process in any of its stages,—or any of those circumstances which produce irregular contraction of the uterus, are, singly or combined, the causes of inversion.

*Treatment.*—When the uterus is inverted only in a slight degree, the reduction may be accomplished with great ease, and the attempt should be made as soon as it is discovered. As the fundus uteri has not, or only slightly, passed through the os, the placenta cannot wholly protrude through this orifice, and, consequently, the fundus should be returned before the placenta is separated. For, if an attempt were made to detach the placenta, the operation must be slow, uncertain and incomplete, and the danger of hemorrhage incurred, or a greater degree of inversion produced. When the hand is introduced through the os uteri, the fingers should be slightly bent, so as to form a kind of crutch, to carry up the fundus, which sometimes rapidly springs up. The placenta is now to be separated, and the hand retained until the uterus contracts.

In the treatment of this accident, (great inversion,) the great object to be constantly kept in view is to attempt the re-inversion, as soon as possible after the occurrence. But in general, the placenta adheres to the inverted organ, and the question is whether it should be separated or not before or after the reduction. It is an important point to settle, especially as there is such a difference of opinion upon the subject. * * * The dread of hemorrhage is the reason assigned why the placenta should not be first detached, but the writer trusts that the cases he has adduced, and the references he has made, are sufficient evidence to the contrary. In no case has this dreaded effect been induced, or even aggravated, by a *complete* separation of the placenta. The uterine vessels are as effectually constricted under this accident as when the organ is in its natural situation, if the placenta be entirely detached; and flooding is produced here, in the same manner as in ordinary cases, by a partial separation or disruption. As the greatest disadvantage arises from failing in our first attempt, it is the more necessary that every impediment should be removed, so that we can proceed with the greatest chance of success. By delay, the organ becomes less fit to bear the operation, not only from the increased size of the fundus and the contraction of the os, but also from the increased sensibility and irritability which it has acquired, even previously to its becoming actually inflamed. The attached placenta must increase the obstacle, because the fundus cannot be so freely and sufficiently compressed. The result of free manipulation would lead to partial detachment and disruption, and consequently to flooding. By detaching the placenta, great advantages are gained; the bulk of the part is diminished, the operator is enabled further to reduce the size of the fundus itself by compression; and he has more freedom to judge of the changes he has effected.

When the placenta is detached, our next object should be to attempt the reduction of the general bulk of the tumour, by compressing it. We are indebted to Mr. C. White, for this method. The plan recommended by some writers, to push the fundus directly upwards, should not be adopted. There are strong reasons to think that the fundus is, after the os uteri, the most irritable part of this organ. When the accident has existed a short time, pressure upon this portion induces pain, bearing down, and hemorrhage; but the body may be taken hold of and compressed. If we could press the fundus upwards, and thereby dimple it within itself, we should find ourselves opposed by a double inflexion; for the body would be grasped by the os uteri, and the fundus would be within the body. It is obvious that our force should be directed so as to act upon the angle of inflexion, or where it turns into itself."

It will be found that the tumour will freely pass through the os externum, and, as only one hand can be admitted into the vagina, the chief compression should be effected whilst it lies externally. And, as the upper part of the vagina descends along with the uterus, no real effect can be produced until it is made tense by carrying this organ upwards. When it arrives at this point, resistance is met with, but, by keeping a steady pressure upwards, the inflected portion of the cervix then yields, and it gradually recedes, followed by the hand of the operator, until the reduction is completed.—*Dublin Journal of Medical Science.—Dunglison's Intelligencer.*

—

3. *On the Nature and Treatment of Nervous Exhaustion, and the deleterious effects of Mercury.*

To the Editor of the Lancet.

Sir:—Allow me to enter, on the pages of the leading Journal of medical science, my earnest protest against the daily and destructive practice of treating the effects of nervous exhaustion and general torpor of the system, as dependent on disease of the liver, and as requiring, in consequence, the administration of mercury. From the conviction resulting from experience, I can positively affirm that the sufferings of most of those persons who are said to be affected with disease of the liver are produced by mercury; that, by the frequent use of mercury, those sufferings are protracted for years, though still said to arise from an imaginary hepatic disease, enduring for ten or even twenty years; and, finally, that by the frequent and long-continued administration of mercury all hope and chance of a recovery, which time alone would often effect, are utterly extinguished. Some practitioners are in the habit of treating all obscure disease as disease of the liver; a pain in the right side or shoulder (often rheumatic, or traceable to tenderness in the spine, especially in hysterical women,) they consider as perfectly diagnostic; with them the heroic, the omnipotent specific is mercury,—the only salvation, salivation. With this introductory protest, I beg leave to offer a few practical observations on the ætiology and rational treatment of nervous exhaustion.

Anxiety, affliction, dissipation, fatigue, exhaust the nervous power: a foul atmosphere, especially when contaminated by the effluvia from drains and rivers, exerts the same exhausting influence: it is often easy to discern, from the general aspect of the inhabitants, that the habitation they dwell in is infected by the effluvia from putrifying animal matter. The countenance of the sufferer from nervous exhaustion indicates langour, melancholy, depondency; the eyebrows become distorted from their harmonious level, and give to the countenance a peculiar expression of wretchedness; in healthy youth the eyebrows are traced upon the same horizontal line; they express serenity; in healthy age the left eyebrow is generally depressed, and denotes reflection and placid meditation; but whether in youth or age a depression of the eyebrow indicates anxiety and distress, nor, indeed, can the eyebrow be thus voluntarily drawn down without exciting a corresponding feeling of uneasiness.

From exhaustion of nervous power, the liver necessarily becomes torpid; it participates in the general torpor of the system, which may present no prominent symptom; but the symptoms of a torpid liver are so remarkable as often to attract the whole and sole attention of the practitioner: the slow or feeble pulse, the inactive state of the digestive organs, the deficient secretion of bile, all tend to confirm the favorite and general notion, that there is a disease of the liver; the specific and reputed remedy is prescribed as a matter of course: the employment of mercury is apparently justified by its immediate effects; the stimulus it affords to the system generally, and the liver in particular, removes, for a time, the more obvious symptoms of disease; but again and again those symptoms recur, and at each recurrence mark the increasing debility of the patient; the liver at last will scarcely act, excepting when under the unnatural influence of mercury; but the stimulus which excites the liver exhausts the brain and nervous system; mercury is, therefore, a dangerous and deceiving poison to those who are already fainting and sinking beneath the pressure of accumulating evils; for a time it confers relief, but eventually becomes the cause of much of the misery that embitters the existence of the nervous invalid; misery compounded of gloomy thoughts, abject fears, the dread of madness, the weariness of life, that tempts him to die the death of the suicide, in hope of rest. The sufferer from nervous exhaustion will often detect the administration of the smallest dose of mercury by the almost insupportable langour it produces; a mercurial dressing applied to an ulcer will cause, in many, neuralgic affections: the deep and frequent sigh, and the tottering pace of a drunken man, evince the effects of large and frequent quantities. The eagerness of the public to embrace new systems, the extraordinary appetite for universal pills, is to be accounted for by a growing antipathy to mercurialists and phlebotomists, and by a natural disinclination to protracted illness; the homœapathic system possesses the negative merit of doing no harm.

Nervous exhaustion is associated with a peculiar condition of the system. I may here remark, that the particular conditions of the body which engender or modify various diseases are little regarded by medical men; there is, for instance, a rheumatic condition, the existence of which is manifested by the tongue, and which, if early attended to, may be easily corrected by dietetic measures, by medicines which act as chemical correctives, and by withholding the elements of rheumatic disease, abundantly contained in animal food and fermented liquors; this rheumatic condition being neglected, leads to violent and destructive inflammation of organs; the organ attacked will be the one which is congenitally the weakest, or which has become so by long-continued excitement. The young practitioner will soon learn to dread the rheumatic fever that attacks an individual who has long struggled with difficulties; the brain will certainly be involved. A vast number of diseases may be termed rheumatic as arising from, or influenced by, a rheumatic condition of the body; there are rheumatic bronchitis, rheumatic diarrhœa, rheumatic inflammation, following injuries or surgical operations, &c. &c. The condition of the body, with which nervous exhaustion is associated, is essentially anemic. I shall venture to term such a condition a leucotic condition, or leucosis, closely resembling, if not identical with, chlorosis; it is a condition common to men and women, to infancy and age. A leucotic condition of the system is distinguishable by many external and visible signs, but especially by the appearances of the tongue: the tongue is pale, like a piece of raw veal, often coated, moist, deeply marked with impressions from the teeth, flaccid, often tremulous, almost bloodless, a muscular structure without power; it is a type of the system generally; the whole system is bloodless; the muscular system powerless; the countenance pale, as from the effects of lead; the lips blanched; the feet are continually cold; the urine pale, sometimes turbid, not often red, when red, only for a short period; of course, there are different degrees of the affection, sometimes it requires a practised eye to discern the earliest degree, as shown by the pallescent and slightly-swollen tongue. The leucotic condition, it is important to remember, is especially to be recognised by the appearances of the tongue; the body may be pale and emaciated, yet the tongue and lips be red; in such a case there is nervous irritability,—not nervous exhaustion,—the treatment must be widely different.

When there exists a leucotic condition of the system, the liver is generally inactive; there may be congestion of the liver from deficient nervous impulse to secretion; but this inactive and congestive state of the liver is sympathetic, and of secondary importance; it will generally disappear as the constitution acquires power; sometimes, when milder means prove unavailing, a dose of mercury may be required to excite an immediate disposition to healthful action, but by a frequent repetition of the dose the power to act is gradually lessened, and, at last, destroyed.

Persons of leucotic habit are very numerous in populous cities; they are affected like the rest of mankind with diseases of specific character, but the specific treatment often aggravates the pre-existing condition of the system, and proves fatal to the patient. No error is more fatal to them than the prevalent one of assigning fearful names to trivial symptoms; if the name be fearful, still more fearful is the treatment; to them the term inflammation, from the treatment it is supposed to necessitate, is far more destructive than inflammation itself; an unfavorable prognosis is sometimes confirmed, not so much by the natural termination of a disease as by the result of the treatment.

A leucotic condition predisposes to the attacks of debilitating epidemics; it is observable in the stout and bulky, as well as in the thin and slender; it often leads to enlargement of the heart from flaccidity of the parietes, to various ramollissement, to dropsical effusions, to enlargement of the spleen in infants; when the last-mentioned disease is detected in an infant, it will generally be found that the leucotic condition is evident either in the father or the mother, or in both.

The leucotic subject often suffers from distressing headaches, especially of the upper or hinder part of the head, from deafness, from deficient sensibility, from bronchitis, especially in infancy and age, from pain in the precordial region, and palpitation of the heart; these diseases are often rendered tedious or fatal by cupping and bleeding, by purgatives and expectorants.

*Treatment of Nervous Exhaustion.*

Patience and perseverance must characterise the judicious treatment of nervous exhaustion; no remarkable benefit can be suddenly effected; the very attempt is productive of injury; the patient must be treated, not by partial, but by general measures, and of these the moral are as important as the physical or medicinal. Tranquilize the mind, still the troubled heart, engage the attention and interest of the patient by amusement however trivial; if the mind be refreshed, the impulse to improvement is already gained. Excitement is destruction; the excitement of irrepressible anxiety and anguish has but one antidote, the oblivion of death; poverty soon consigns the sensitive victim from the poor-house surgeon to the tomb. Dietetic excitement is pernicious, whether produced by too highly anamalized food, or by fermented liquors; the strengthening system, as it is commonly denominated, consisting in the administration of indigestible quantities of animal food, eggs, jellies, bread and milk, and large quantities of beer, wine, or spirits, only excites and irritates; debility and inability are often combined, and claim an equal regard; the inclination of the patient may safely direct the choice of food; when the tongue is pale and flabby, the patient is generally fond of salt and of red meats, and may be allowed to partake freely of both; salt is corrective of the morbid condition. One of the best cordials not included in the Pharmacopœia is Guinness's bottled stout, in limited quantity. When the tongue is coated and appetite defective, when the mercurialist would prescribe his favorite anti-bilious pills, then a farinaceous diet is most beneficial, and is generally manageable to the patient; food should be taken, or abstained from, as the tongue is clean or foul; a clean tongue denotes complete digestion, as a clear fire denotes perfect combustion. When there is instinctive loathing of solid meat there is no wisdom, though often much self-congratulation in deluding the palate with beef-tea, milk, &c. To the success of the measures adopted, gentle exercise in the open air, and on a level surface, is indispensable; irregular and distressing exercise, such as running up and down stairs, shaking beds, &c., though often recommended, is worse than useless. Regulated exercise should be attended with perfect rest, with rest in the recumbent position; the spinal column should be placed in the position of absolute rest, the horizontal position; it is difficult to acquire strength, it is easy to reserve it; much of the sufferings of nervous invalids would be avoided, if the recumbent position, as in former ages, were more fashionable than the sedentary. The feet of the patient *must* be kept warm, an imperative injunction, which must be fulfilled, however great the difficulty.

The medicinal treatment must be chiefly regulated by the appearances of the tongue: when the tongue is coated with a yellow, greenish, or cream-like substance, the edges being pale as usual, then gentle aperients should be regularly given, such as aloes, colocynth, or senna tea, all of which, like mercury, exert a peculiar influence on the liver, without its evil consequences; irritability is best allayed by minute doses of tartar emetic, which do not debilitate, given at bedtime. Sometimes a very small quantity of opium is serviceable; an excellent draught is formed by a combination of small doses of opium, hydrocyanic acid and tartar emetic, (it will be found the more excellent, when there is nervous irritability, with a dry and red tongue); opium is generally a very dangerous medicine in cases of nervous exhaustion, increasing the general torpor. Epsom salts, if given at all, should only be given in small quantity, they then produce a tonic effect; in large quantities, they are remarkably debilitating; when the tongue is pale and moist, the extract of aloes will seldom occasion hæmorrhoids; when the tongue is red, especially if red and dry, it rarely fails to produce them; salts, and, indeed, all drastic purgatives, have the same effect; this assertion may easily be verified by experiment.

When the condition of the system is evidently leucotic, when the tongue is pale and flabby, and almost bloodless, like a piece of raw veal, taking an impression from the teeth, then the imperial remedy, the real specific, is iron—iron in any form, not, however, given by handfuls, which disgust and dispirit. Dr. Sigmond, in his interesting and instructive lectures, has stated that iron is not to be given in cases of phthisis; but when phthisis attends a leucotic or chlorotic subject, there is no medicine which so long postpones the fatal termination; the tongue in phthisis is generally the antithesis to the one described; it is morbidly, intensely red; then, indeed, iron is pernicious, and so is the treatment generally followed of supporting the patient's strength by wine and indigestible quantities of stimulating food; the distress, and fever, and cough, are all aggravated, and the progress of the disease accelerated by irritation. The flabby and indented tongue has been considered as particularly indicative of disease of the liver; it is the tongue that should especially preclude the employment of mercury; iron may be given with advantage where the tongue is pale and moist, even though it be coated; gentle aperients may be administered at the same time. Mercury should never be administered when the tongue is clean, and is only cautiously and occasionally to be given when a yellow, greyish, or greenish coating pertinaciously adheres to it, despite all previous efforts to remove it.

Mercury acts most beneficially in all cases where the tongue is covered with a greyish or yellowish coating, which appears to have dyed the tongue, and to be incorporated with it, and when the tip and edges of that organ are red or purple, and parched (such is the ordinary tongue of the drunkard and of the patient suffering from acute bilious fever), the hydrag. c. creta is generally the best form; mercury is productive of minor injury when the tongue is clean, dry, and red; when there is nervous irritability,—not nervous exhaustion,—it will rather irritate than depress; oppression of strength is often mistaken for real debility; the tongue affords the means of distinction.

Mercury is oftentimes an invaluable, an indispensable, remedy, but should never be unnecessarily prescribed: mercury and bleeding are both powerful agents, but he is the most scientific practitioner who, with safety to the patient, can most frequently dispense with either.

Much more might be usefully written, much more *will* be hereafter written, by abler men, on the conditions of the body as influencing disease, on the nature of morbid action, and on the elements of disease; primary causes will be as eagerly investigated as ultimate effects. I remain, sir, your obedient servant, HENRY REES.

45, Finsbury-square, Dec. 18th, 1837.—*Lancet.*

---

4. *Clinical Lecture.* By W. LAWRENCE, F. R. S.

*Encysted Tumors of the Eyelids, containing Hair.*—A few days ago I removed a small tumor, in the case of a young child, from the neighborhood of the external canthus. It was about the size of a horse-bean, forming a colourless elevation, with the integuments covering it loosely, so that they could be pinched up into a fold over the swelling, which was more fixed below. It was placed immediately behind, and a little above the junction of the lids; and was said, by the mother, to have been there from the time of birth. She stated that it was increasing, and for this reason she wished that it should be removed.

In performing the operation, it was found that the tumor, which from external examination might have been supposed to be simply subcutaneous, was covered also by the orbicularis palpebrarum, and that it adhered closely to the external angular process of the frontal bone.—It consisted of a thin but compact cyst, with a whitish glistening surface, and it contained a fatty matter, resembling soft suet, with an intermixture of short darkish hairs, principally in small bundles on the surface of the fat.

Such tumors are not unfrequent in infants and young children, occupying the situation just described. I believe them to be congenital; at least the statement of the mother generally leads to this inference.—Sometimes they are stationary: in that case, if the swelling is small, there is no necessity for operation, as it is perfectly indolent. I am acquainted with a gentleman who has had through life a rather larger growth of this kind; it causes an unnatural fulness near the extreme angle of the eye, not amounting to deformity, and has never been attended with the slightest uneasiness. The affection, as in the case just described, is a cyst containing fat, which is sometimes of oily consistence, sometimes firmer. I have always found short hairs mixed with it in various proportions. Is this admixture of hairs, which resemble those of the eyebrow in length, to be regarded as an exemplification of the principle so frequently observed in adventitious structures, viz: that they resemble in nature that of the textures in which they grow, or that of parts in their immediate vicinity? It is placed under the arbicularis, and adheres more or less firmly to the bone. You must bear in mind the two latter circumstances in operating; make a larger incision than the size of the swelling would seem to require; and pay especial attention to the complete removal of the cyst from the bone.

If a portion of the cyst is left, the wound will not close, and such an occurrence is very annoying to the patient, and considered discreditable to the operator. I saw a young lady, in whom such a tumor as those I have described had been removed from the root of the nose, at the interval between the eyebrows. She was a handsome person, and had submitted to the operation for the removal of what she deemed a blemish, though it must have been very slight, as the tumor was inconsiderable. She was much worse after the operation than before; for the wound did not heal, at least it sometimes scabbed over, and sometimes discharged. A probe introduced into the opening went down apparently to the bone. Having learnt the nature of the swelling, and that the operator had experienced unexpected difficulty in separating it from the bone, I concluded that a bit of the cyst had been left behind, and proposed an incision to ascertain that point, which was readily consented to. I found, closely adhering to the frontal bone, a small strip of the cyst, conspicuous by its white glistening surface, and having a few short hairs on it: this was easily removed, and a firm cicatrix was soon secured.

I once saw a small growth of similar nature, but with an external aperture large enough to admit an ordinary dressing probe, on the bridge of the nose. It was congenital; and the opening sometimes produced a kind of greasy discharge. A probe passed in about a third of an inch. Various applications had been made to the part ineffectually. I slit it open and, found a smooth shining membrane with small hairs upon it, almost imbedded in the ossa nasi. It was difficult to dissect it out completely; but the removal was accomplished, and the part healed soundly.

*Penetrating Wound of the Sclerotica, with Prolapsus of the Iris.*

A child, about five years old, was brought to the hospital, as an outpatient, a week ago, having received a severe blow on the right eye with a stick. The sclerotica was ruptured near the margin of the cornea, the wound being about one-eight of an inch in length. The corresponding portion of the iris had disappeared, so that the pupil, opposite to the wound in the sclerotica, was continued to the circumference of the cornea. The conjunctiva was slightly elevated, as if by protrusion of the missing part of the iris. The accident had occurred three or four days previously, and nothing had been done for it. There was no inflammation or pain of the eye: the pupil was clear, the iris natural, except in the points already mentioned, and vision seemed unaffected. The case required nothing further than care, with a little medicine and cold lotion. We saw the child since, the eye remaining in the state already described, and ascertained clearly that vision was unimpared.

Penetrating wounds of the globe seldom terminate so favorably as in this case. They generally produce, more especially if they should be lacerated, as in this patient, serious inflammation, which involves the internal tunics, and is thus likely to destroy or injure sight. To prevent or to lessen this kind of mischief, active antiphlogistic means should be resorted to immediately after the accident, or as soon as the slightest indications of inflammation are observed. Again, a blow on the eye, without producing any wound or visible injury, may impair or destroy sight, by causing concussion of the retina. I use the word concussion here, as in injuries of the head, to denote a species of injury not produced by violence directly applied to the part, and of which indeed we do not know the exact nature, although it is capable of producing the most serious effects. I lately saw a young gentleman in whom complete amaurosis had ensued from a blow in the eye, not followed by any visible injury.

When the sclerotica is wounded, there is commonly an excessive partial dilatation of the pupil opposite to the injury, which may proceed, as in this case, to actual disappearance of the pupillary margin. This must be ascribed to injury of the corresponding ciliary nerves. Such wounds are frequently attended with effusion of blood into the anterior chamber, behind the iris, or in both situations. This indicates that the violence has been serious; and wound or rupture of the sclerotica, combined with such effusions, generally terminates in loss of vision, either by concussion of the retina, or by inflammation of the internal tunics coming on slowly after the accident, and terminating in atrophy of the globe.

I remember, however, another case of complete recovery, after a penetrating wound of the sclerotica, which I mention to you, because such favorable results, although rare, may prevent us from pronouncing too alarming a prognosis. A boy at school, ten years old, received a blow on the eye, near the external angle, from an arrow, with a pointed nail fixed to its end: it was thrown towards him, and did not strike the globe with great force. He was immediately brought to me in a chaise, from a distance of fifteen miles, where the accident had happened. The master, who accompanied him, informed me that he examined the eye immediately after the accident, that he found the pupil greatly dilated, and that the boy could not see. There was a wound of the sclerotica, a short distance behind the cornea, shaped like a leech-bite, but larger: the pupil was round, clear, and larger than that of the opposite eye. I was pleased to find that the patient could see, though imperfectly; and I was unwilling at that moment to make the trials necessary for ascertaining the exact amount of vision. I directed leeches, cold cloths to the eye, aperient medicines, low diet, and rest in bed, strong light being excluded from the room. I saw the patient again in ten days, when the eye was, as it had been in the interval, free from all inflammatory redness; the iris, the pupil, and the motions of the former, were perfectly natural, and vision was unimpaired. The recovery in this case was permanent; the injury of the sclerotica being still visible. Wounds of this structure do not unite, as we see in the punctures made in couching.

*Iritis combined with Scaly Syphilitic Eruption.*

I have seen iritis occurring in conjunction with various forms of syphilitic eruption. If asked with which of these it is most frequently conjoined, I should have said the scaly; although Mr. Carmichael, whose experience has been so extensive, has represented it as almost confined to what he denominates the popular form of venereal disease. I mentioned to you in a former lecture, a case of acute syphilitic iritis, with scaly eruption, and ulceration of the fauces. There was general discoloration of the iris, with a mass of lymph effused near the pupillary margin; contraction and irregularity of the pupil, with vision so much impaired, that the patient could not distinguish even capital letters. The treatment consisted, as I then mentioned to you, in abstraction of blood, by cupping on the temple, to sixteen ounces, and the administration, every six hours, of two grains of calomel, with one-third of a grain of opium. Subsequently, the moistened extract of belladonna has been applied on the brow daily. The mercury has acted considerably on the system, producing ptyalism; and the influence on the ophthalmic affection, and the other symptoms, has been proportionably decisive and rapid. In seven days, the effused lymph is nearly absorbed, the iris has regained its natural color, the external redness has nearly disappeared, and the patient can read small print; the adhesions of the iris have not given way, and the organ has not perfectly recovered, although the mercury has been discontinued, and will, probably, not be resumed. The eruption has faded, and the throat is recovered. The antivenereal powers of mercury seem to me to be shown most unequivocally in ulcerations of the throat and mouth; for although the remedy produces, as it did in this case, inflammation and ulceration of the gums, it cures the syphilitic ulceration of the contiguous membrane, thus appearing in the double light of bane and antidote, in reference to two parts of one continuous membranous surface.

W. Pratt was admitted May 25, with gonorrhœa, scaly eruption, superficial ulcerations of the fauces, and pains in the limbs. On account of the latter, which were severe, hydriodate of potash, in the dose of five grains, three times daily, was administered in the compound decoction of sarsaparilla. It acted with its usual good effects; and the patient felt himself so much relieved, that he left the hospital, by his own desire, on June 14. He returned on the 21st, with acute iritis of the right eye. There was a zone of pink redness in the sclerotica round the cornea; the iris was discolored, dull, and dark throughout; the pupillary margin, and the lesser circle, being covered by a fur of dull brownish red lymph. The pupil was irregular, vision greatly impaired, and there was great pain in the frontal region, particularly during the night, completely preventing rest. Twenty ounces of blood were taken from the temples by cupping: two grains of calomel, and one-third grain of opium, were given every six hours. On the 23d, twelve leeches were applied round the eye: and now (June 26) recovery is proceeding rapidly, under a decided, but not excessive, mercurial influence.

In —— Richardson, we have another example of iritis with scaly eruption. There was general discoloration of the iris, without any effusion of lymph in distinct masses—condylomata, as some of the German writers have called them. Although these effusions are seldom seen in the adult, except in syphilitic iritis, they do not always take place; perhaps they are not met with in the majority of cases. The pain in the frontal region was very severe, particularly at night, and we employed for its relief frictions of mercurial ointment and opium on the part, six grains of the former, with two of the latter, which seldom fail to relieve this particular symptom. The treatment was the same, in other respects, as in the two preceding cases; and this patient is recovering favorably.

Thus you see that we have in the hospital at the same time three cases of syphilitic iritis accompanying the scaly form of eruption.

*Strumous Ophthalmia.*

Mr. Lawrence next adverted to two cases of this complaint, which had been some time in the hospital, and were now completely recovered, under a mode of treatment which he generally finds successful. The most striking symptom of the complaint, intolerance of light, existed in both to its greatest extent; indeed, it might rather be called total inability to bear the light in any shape; for these children lay in bed under the clothes, either with the face resting on the bed, or the hands pressed on the eyes. The face was red and excoriated from the pressure and from the scalding tears, while the physiognomy had the peculiar expression produced by the spasmodic closure of the eyelids, and the combined contraction of all the surrounding muscles, for the purpose of shutting out the light. To get a clear view of the globe was impracticable; but the eyes, as far as they could be examined, presented very little appearance of disorder. In these strumous subjects the occurrence of disease in a new part relieves that which was previously suffering. In imitation of this natural process, the tartar emetic ointment was rubbed on the nape in both patients, so as to produce and maintain a considerable irritation; the sulphate of quinine was administered internally; the bowels were regulated by small doses of rhubarb; animal diet was allowed, when the appetite indicated the necessity for it; and the suffering organs were occasionally fomented with tepid water. Under this treatment, both cases have recovered: the children can now open their eyes, and bear the light as well as any body, and we find that no unfavorable change has occured in the affected parts. The patients are at the same time improved in general health. In one of them, the pustular eruption caused by the ointment spread over the body, with considerable, but temporary constitutional disturbance.—*Ibid.*

### 5. *On Inhalation in Tubercular Phthisis Pulmonalis.*

To the Editor of the Medical Gazette.

Sir:—The favour of your insertion of the following further observations, on the subject of inhalation in Tubercular Phthisis Pulmonalis, will oblige, sir,

Your obedient servant,

Charles Scudamore.

Wimpole Street, Feb. 10, 1838.

Three years have elapsed since my last communication in the Medical Gazette, of the successful treatment of some cases of phthisis pulmonalis, in which the inhalation of iodine and conium formed a material remedy. I hope, therefore, that a concise summary account of my further experience in this lamentable disease, and in chronic bronchitis, may prove an acceptable offering to the profession. That it has been my fate to meet with a much larger portion of failure than success, in phthisis, cannot fairly be made the reproach of my plan of treatment; for it has happened to me, for the most part, that I have not been consulted till the advanced stage of the disease, when the lungs having become extensively disorganised by the spread of tubercles almost universal, by cavities the consequence of the softening process, and often with the complication of ulcers in the intestinal canal, my task could only be that of studying to alleviate symptoms, and to soothe, by tender care, the last weeks or days of life.

It not unfrequently happens with consumption to be overlooked in the early stage of the disease, when probably attentive appropriate measures might be attended with happy results. The danger is serious, however delayed the consummation of the event, when tubercles have began to infest the lungs. But from the fear and anxiety of friends inducing a flattering rather than a true view of the case, with a reluctance to listen to any voice that gives a warning of danger; and from the tenderness probably more than the better judgement of the medical adviser, which makes him join in the delusion, it follows, unhappily, that the early indications of dangerous disease are not detected or sufficiently regarded, and the most valuable period for giving assistance is lost; perhaps irrevocably. I have known very many instances exactly exemplifying this statement. The consoling opinion has been pronounced, "that at present the lungs appear to be safe," or that, notwithstanding the evident delicacy of constitution, and necessity for care, yet "that there is no reason to consider the lungs at all affected;" while at this very time tubercles do exist, and consequently danger is established.

It will be said, that in the commencement of tubercular phthisis, there is much obscurity in the symptoms, and that we cannot discover by auscultation whether or not tubercles exist, as they may be so minute and disseminated, or so very partially clustered, as not materially to interfere with the vesicular respiration. I freely admit the insufficiency of the stethoscope to detect the early state of the tubercular disease, on many occasions at least; but there are several circumstances largely instructive.

If the patient have lost strength and flesh, without apparent cause; have recently become short-breathed on slight exertion, especially on making the least ascent; have more or less of short dry cough, a quickness of pulse, night-restlessness, and perhaps some dulness on percussion, here and there, in the upper parts of the chest, we have surely great reason to fear that there are tubercles formed. If other members of the family have died from consumption, the suspicion is painfully increased.

This is the most important period for the adoption of a systematic plan of treatment, and the one at which I should have the most confidence in the efficacy of the inhalation of iodine and conium. But a suitable treatment embraces many other points of management.

I believe I may state with truth, that when the symptoms become so conspicuous as to receive medical notice, the most general principle of practice has been to seek, as the *summum bonum*, a retardation of the pulse, and that by means of sedative medicines, the use of digitalis especially, with a slender, light, and cooling diet. Sometimes this is restrained to farinaceous food, milk, vegetables, and fruits. I apprehend that only in the very acute cases of consumption, which occasionally happen to young persons, is such a mode of treatment at all proper. When it is not forbidden, as in such cases, by the high degree of hectic fever so prevailing as to be almost continued, it appears to me of great importance to sustain the powers of the constitution by a very generous and supporting diet, with some porter; and with or without the addition of diluted wine, instead of aqueous beverage.—I also make choice of tonic alterative medicines rather than those of a debilitating nature; soothe the nervous system at night by anodynes and sedatives, and employ such mode of external counter-irritation as may be best suited to the individual case, for this must be varied. Some patients are so much lowered and distressed by blisters, that they become inadmissible. I find the oil of croton liniment often to be very useful, and much less inconvenient in its effects than the tartar emetic ointment. I attach importance to the daily ablution of the chest with a mixture of purified acetic acid, Eau de Cologne and water, substituting for water an infusion of tannin, when perspirations are excessive. All the general means of Hygeia are of course to be pursued. Lastly, and what, in my mind, is a most important link in the chain, I make use of the inhalation of iodine with conium, according to the formula stated in the second edition of my work,* and in the Medical Gazette, vol. viii. p. 157. And here I will take occasion to make some further remarks on the use of this remedy.

It is of the utmost importance that all the medicines used for inhalation should be quite pure, and that the tinctures should be saturated with their ingredients. If the iodine be in the dry state it requires an equal portion of iodide of potassium, that it may remain in permanent solution in the mixture; *for* "three" grains of this salt, expressed in my formula, *read* "five:" and according to the strength of the solution, so the proportions used are to be smaller. It is better always to add

* "Cases illustrating and confirming the remedial power of the Inhalation of Iodine and Conium in Tubercular Phthisis, and various disordered states of the lungs and air-passages."

the conium at the time of using the inhaling. At the temperature of 90 deg. the volatile properties of the iodine are given off very sensibly; but the conium requires more heat, and that of 120 deg. is not too much for the iodine. This degree, therefore, I most recommend; or if the patient have not a thermometer, let the instruction be, to put the water into the inhaler (first warming it a little to prepare it) quite as hot as the finger can bear without pain. The inhaler should be kept immersed in rather hotter water during the process. A good glass inhaler also is a material consideration. If it be small, and the tubes too contracted in the bore, the difficulty of inhaling would be great to the invalid, whose respiration is easily embarrassed; whereas, with a fit apparatus,* the process is perfectly easy, and not fatiguing.

It would be inconsistent with my present purpose to enter at length into the *ratio medendi;* for which and other particulars I beg leave to refer the reader to my publication.

The Case V., detailed in my second edition, very clearly demonstrates the useful power of the inhaling treatment. This gentleman was labouring under the well marked symptoms of pulmonary tubercles, much increased beyond the usual degree in which he had been for some time affected, and so as to excite immediate alarm for his safety. He recovered in so satisfactory a manner under my care, that he was enabled to pass the two following years at his seat in Scotland, and to enjoy the pleasures of his gun and hunting. He wrote me the most cheerful accounts, and described himself as perfectly well. I received a similar statement from his friends. He was of the most active disposition, too convivial, and passionately fond of field sports. At the end of the second year he became quite careless of his safety, often exposing himself to the most unfavourable weather, and frequently committing great excess at the table in wine and mixed spirits.—Thus he renewed in the most active manner the disease which had been so satisfactorily controlled. I learnt that in a short time hæmoptysis occurred, attended with urgent cough; this was followed by a severe attack of influenza; and when, at a late period, I saw him in London, his situation was hopeless. A large excavation had formed in the upper part of the right lung, and a smaller one in the left. He was much emaciated, but he combated with his disease for a considerable time, till at last the strength of his constitution yielded.

I allude to this case for the purpose of shewing, as it appears to me, the beneficial influence of the inhalation in the first instance, on the tubercular disease. Had the patient led a life of proper care; it is reasonable to suppose that the recovery which he had obtained might have been lasting.

I wish now to refer to some of my other published cases; and first to those which will be found in the number for February, of the fifteenth volume of the Gazette.

J. A. Case I. He had remained comfortably well from November 1834, till the period of influenza,† in the beginning of 1837. He

* The best inhalers which I have seen are to be procured at Garden's, chemist, 372, Oxford street; and Perkin's chemist, 147, Oxford street.

†How severely did this epidemic test the vital power of all delicate constitutions, and more especially all those affected with pulmonary disease, or predisposed to phthisis pulmonalis.

was violently attacked with the worst symptoms of the epidemic, and fell a victim in a short time.

*Case* II.—This gentleman continues to enjoy very excellent health, has married, and established himself in life.

The subject of Case III. has been free from all returns of disease.

*Case* IV.—This gentleman has acquired so settled a state of health, that within the last few months he has married.

*Case* V.—This young woman informs me that since the period of my last report, she has very seldom had occasion to resort to the inhalation; but that when she has done so, she has never been disappointed in its good effects.

Of much the larger part of the cases related in my second edition, I could make an equally favourable report. I shall advert only to some of the most important examples.

*Case* I. (2d series.)—This lady has continued in the enjoyment of very comfortable health, possessing good strength and spirits up to the present time, with the exception of having suffered from influenza on each occasion of the epidemic occurring. It is most satisfactory to consider that her remarkable recovery was so perfect as to admit of such a trial of its stability.

*Case* VIII.—I have received the most agreeable accounts of the continued health of this gentleman.

*Case* IX.—This young woman has continued to enjoy comfortable health.

I will now offer an outline of a small part of my more recent experience in consumptive cases:—

A lady, aged 22, mother of three children, consulted me in April 1835. She had been falling off in health for a year past. Her youngest child was fifteen months old, and she had nursed it for twelve, but with difficulty. She related that she had within the last few months lost flesh and strength very rapidly; that on six or seven occasions she had coughed up small quantities of pure blood, but latterly it had only appeared occasionally, streaking the expectoration.

At my visit I found her suffering from harrassing cough, with the inspiration easily hurried by slight exertion, and a distressing sense of restraint over the chest. She had daily hectic fever, and severe night perspirations; the pulse upwards of 100; the animal heat 100 deg.; the appetite was lost, and the spirits much depressed. The sputum was considerable in quantity, puromuculent in appearance, offensive in odour, and slightly streaked with blood. The signs by auscultation were, much mucous râle on each side, but on the right especially, and there mixed with the sibilant. In this part also the voice was very resonant, and on percussion the sound was dull. I considered that there were tubercles, but that no softening had taken place.

It had been thought proper to keep her on very low diet; and it had been candidly stated to her friends, that as further trials with medicinal treatment could not in all probability render any benefit, it would be most expedient to try a change of air, and trust to that alone.

Without delay I prescribed the inhalation of iodine and conium; gave tonic and alterative medicines; used counter-irritation by means of oil of croton diluted with spir. ammon. comp. over the most affected parts of the chest; and elsewhere it was washed daily with a lotion of infusion of tannin, purified acetic acid, and Eau de Cologne, slightly tepid.

She experienced very sensible relief from the inhalation, and in a short time was enabled to lie down in bed comfortably on either side, from which she had been long prevented, and the embarrassment of her breathing on exercise was most satisfactorily relieved; she could take a considerable walk without inconvenience.

This lady quite recovered her health at the end of four months, and has since continued well, a period of almost three years.

A gentleman, aged 25, tall and slight, of consumptive family, and had recently lost a brother from consumption, when under the influence of mercury, exposed himself to wet and cold, and in consequence was attacked by acute rheumatism. Having, after a long period, regained his health, he joined in the sports of the field; and one day, when much heated and in a perspiration, forded a river, and kept on his wet clothes for some hours. Catarrhal fever and cough quickly ensued, and in a short time, his state, as described to me, was that of a person labouring under the first symptoms of consumption. He took an early opportunity of visiting Torquay, and resided there some months, but made no progress towards health, although he had diligently followed the advice of his medical friends. One acquaintance had recommended him to consult me; but another, and he was medical, disuaded him, assuring him that inhaling iodine would do him serious mischief. He passed through London on his way to the continent, Rome being his destination; and consulted a physician, who, with other means, prescribed the inhaling of creosote, which he tried steadily. It did not disagree, but proved of no benefit. He was exposed to many inconveniences in travelling, and at one place was detained in quarantine on account of cholera. His disease increased so much before and after his arrival at Rome, that his state became truly alarming; and his debility and emaciation had reached a fearful height. It occurred to him that, having possession of my book, he might adopt the treatment recommended in it, under the guidance of an English Physician whom he selected.* He went afterwards to Naples, and followed up the treatment there. The terms of approbation in which he expressed himself, when he described the extraordinary benefit which he derived from the inhaling of the iodine with conium, were enthusiastic; and he declared himself to have amended from the first moment. In the account which he gave me of his case at that time, when at Rome, he stated that he had daily two paroxysms of hectic fever; suffered most severely from cough, attended with an offensive expectoration, which was frequently coloured; shortness of breath; loss of sleep at night; copious perspirations, and other bad symptoms. The hectic fever was immediately controlled by the inhalation, and the respiration remarkably relieved.

* This was Dr. Thompson, whom I have just now had the pleasure of seeing. He informs me that when he first saw this gentleman, he was evidently labouring under tubercular phthisis, and having a cavity in the upper part of the right lung. After some interval he consented to the use of inhalation, although with some reluctance. Recourse was soon had to the mixture of iodine with conium; and Dr. Thompson confesses himself quite satisfied with the benefit which the patient received from the inhaling.

I found him still an invalid, but, according to his report, surprisingly increased in flesh and strength; and he was in good spirits. His weight, he said, was within seven pounds of what it had been formerly in his best health. By auscultation and percussion I discovered evident signs of obstruction at the upper part of the right lung, and slighter in the same situation of the left. There was no indication of cavity, but the mucous membrane of the bronchial tubes was not in a healthy state, and there was still cough. The pulse was moderate, and the animal heat 97 deg.

I recommended that the inhalation should be resumed; internally sarsaparilla, with small doses of the iodide of potassium; and externally, the daily use of ablution with the lotion of which I have before spoken, together with friction; that he should live well regulating his diet, and lead a life of care.

The following statement of a young lady, who was evidently suffering from the early symptoms of tubercular irritation, and whose sister died from consumption, is, I think, sufficiently interesting to be related. I will give it in her own natural language:—

"At a period when I was suffering from one of my usual attacks of painful constriction of the chest, accompanied by general depression of spirits, and sometimes with shivering, followed by heat, shortness of breathing, and a troublesome cough, I was recommended by Sir C. Scudamore to make trial of his iodine and hemlock inhalation; and I cannot too strongly express my gratitude for the effects which it produced in relieving all my symptoms. After several times inhaling, the sense of soreness seemed healed, the pain removed, and I felt more expansion and strength of chest than I had experienced for a long period. To express more strongly my feeling from the inhaling, I should say, the effect was like the opening of valves that had long been closed."

Were space convenient, I should relate the cases of a gentleman, aged 54; of a young lady, aged 20; and of a medical practitioner, aged 30; in which the most unequivocal symptoms of tubercular disease were strongly developed; in which there was every threatening of danger; and in all of them I was happily quite successful.

I prefer, however, to substitute the case of Dr. Davidson, of Setham, near Forfare, subjoining the communication with which he has favored me of other cases, and requesting that his letter may be copied *verbatim:*—

*Case of Dr. Davidson.*—About the 1st of September (1836,) I was seized with catarrh, which continued with slight aggravation till the 1st of October, when I was affected with severe pain in the left mammary region, and all at once the sputa, which had usually been from one to two ounces in quantity in the twenty-four hours, assumed a puriform appearance. By general and topical bleeding the pain of my side was relieved, but soon returned in a slight degree, and was then removed by a blister; yet I felt uneasiness behind the sternum. I was confined to my bed, and my strength was much reduced. My pulse, which in health is 60, was then betwen 80 and 90.

He proceeded to state he was apprehensive of phthisis, and wished my opinion as to the treatment of his case. In his second communication, in January 1837, he reported that he had inhaled the mixture of iodine with conium, and taken the medicines which I had prescribed, with much benefit; but that a severe attack of the epidemic influenza had reproduced his symptoms, and compelled him to suspend the treatment for a short time. In his third letter, Jan. 22, 1838, he relates, that after an interval of three weeks from the time of being attacked with the influenza, he had returned to my method of treatment, of which he thus speaks:—"I resumed the inhalation, and with the same happy effect as formerly, for in the course of about three weeks the purulent expectoration gradually subsided, along with the cough and pains of the chest, and by the first of April I was so much improved in health and strength, that I was able again to fulfil my active professional duties. It is proper to mention that, while using the inhalation, I took at bed-time a small dose of the solution of acetate of morphia, and once or twice in the day a few drops of acid. hydrocyan, in an effervescing draught. Over the site of pain in the left mammary region, I kept up counter-irritation from time to time, by emplast. cantharid., or by ung. antimon. tartarizat. I regulated the bowels by a powder composed of pulv. rhei. carbon. sodæ, p. zingib. et sulph. ferri.

My much lamented friend, the late Dr. Mackintosh, of Edinburgh, as well as others, assured me that the left lobes of my lungs were rather emphysematous, which renders me somewhat more liable to catch cold; but with that one exception, thanks to Divine Providence and your kind assistance, I am now in as good health as I have been for many years past. With two exceptions, I have not even had a catarrh during the present winter. These attacks were slight and of slight duration, although I have been daily and nightly exposed, in the discharge of my professional duties. Having given you these particulars of my own case, I will now offer you the relation of a few other cases, in which I have given the inhalation of iodine and conium a fair trial.

January 7, 1837, I was requested to visit Mr. G——, aged 66, parish school master, of Kirkden. My assistant stated that he complained much of tightness across the chest, pain in the left mammary region, severe cough, with muco-purulent expectoration; bowels costive; tongue dirty towards the root; fauces red and tender; pulse 100, and the body weak and much emaciated. My assistant attended the patient, and treated his case with much skill, till the 3d of March, when I visited him myself, and found that from the usual remedies which had been tried, he had derived no benefit whatever. Upon auscultating the chest, I found the vesicular murmur faint on the left side, and marked by bronchial respiration, or mucous râle. The muco-purulent expectoration had greatly increased since he came under our care, and his body was reduced to a perfect skeleton. I recommended the inhalation of iodine and conium, and gave him your work to read. He commenced on the 6th, and inhaled the medicated vapour three times a day till the 1st of May, when he had recovered so much that I made him reduce it to twice; and by the 15th of the said month he gave it up, and resumed his teaching by the 25th, and has continued in full enjoyment of good health ever since. It is proper to state, that with the exception of one small blister, and occasionally a gentle dose of some opening medicine, he used no medicine whatever after he commenced the inhalation.

Mrs. F——, a woman of very delicate constitution, æt. 40, had been complaining from the 9th December 1836. As Dr. M'Donald, my assistant, attended her during the early part of her illness, I am not able to give you the early history of her case. When I saw her, about the beginning of February, she was much emaciated, complained of severe pain in her right side, with tightness across her chest; incessant cough, with copious expectoration, amounting to two English pints every 24 hours. Copious night sweats, with well-marked hectict fever. Pulse 120. The matter expectorated was of a highly purulent nature, and her relations and others who saw her were of opinion that she could not survive many weeks. The stethoscope applied under the right clavicle detected well-marked pectoriloquism, and the respiration absent to a very considerable extent. The parts were very dull on percussion. I prescribed the inhalation of the iodine mixture, &c. renewed the blister, and ordered a wine-glassfull of the mistura ferri composita of the London Dispensatory, to be taken three times a day. The change which was effected in this patient in the course of a fortnight, was quite remarkable. Want of room prevents me from giving you particulars, but I am proud to say that, in the course of one month, her cure was greatly advanced, and, on the 10th of April, she paid me a domiciliary visit, having walked a distance of three miles. I saw her lately in perfect good health.

June 14th, Mrs. S——, a farmer's lady, about 47 years of age, who had been long subject to winter colds and weakness of the chest, requested me to visit her. I found her in bed. She complained of tightness across the chest, with shooting pains betwixt the shoulders. Her cough was quite distressing, and she expectorated, with much difficulty, about ℥x. of muco-purulent matter every twenty-four hours. Pulse 96. Auscultation detected bronchophony in the right axilla; respiratory murmur absent over a great portion of the right side, and very weak over the whole. She had night sweats, and had lost much flesh. I prescribed the inhaling mixture, and iron medicine, all of which she used with much diligence. Being at a distance, I saw her but seldom. On the 20th she expressed herself better, and her pulse down to 86. On the 25th she was visibly improved, and on the 6th July she was able to drive this length along with her husband. I did not see her again until last week, when I had occasion to visit one of her daughters; and I am happy to state that she continues well.

Mrs. Y——, the wife of a very respectable farmer, æt. 34, who had been subject to severe attacks of inflammation of the lungs and pleura, was delivered of a child on the 15th July last. Had a fair recovery, but a cough, which had annoyed her during pregnancy, now became much worse, and her sputa assumed a purulent appearance. Pulse 70, tongue clean, and bowels nearly regular. Complained of severe tightness across the chest. Auscultation discovered that the vesicular murmur was weak all over the chest, and nearly extinct in the scapular and interscapular regions on the left side. On the 5th of August I prescribed the inhaling mixture, which she contined to use, with many of the other auxilliary remedies used in such cases, for nearly two months, but, I am sorry to say, without deriving the least benefit from any one of them, when I considered her case quite hopeless, and for some time past I have adopted a palliative course of treatment only. She is still in life, but ere many weeks elapse she will have closed her earthly account. The now evident issue of her case has both surprised and disappointed me, as I, at the outset, had every reason to expect a favorable termination.

July 30th, Mrs. W——, æt. 27, of a dark complexion and narrow chest, the lady of a minister of the Church of Scotland, in the Highlands came to her father's in this neighborhood, and was placed under my care. She had been mostly confined, since January 1837, with a hard confined cough, which prevented her from obtaining any thing like regular rest in the night; shooting pains in, and tightness across the chest, and great difficulty of breathing; and for the last four months, she had purulent expectoration, to the extent of 5 oz. or 6 oz. every twenty-four hours, with occasional night sweats, and slight accessions of hectic fever at night. Pulse 120. She was very thin, and had much anxiety in the expression of her countenance. Respiratory murmur very faint, and nearly extinct in the subclavia region on the left side. Percussion natural. I ordered the medicated vapour of iodine and conium to be used three times a day; the body to be sponged with vinegar, &c. with the use of the flesh-brush; a blister to the chest, and the mistura ferri comp. to be taken three times a day. In the course of a week her breathing was much relieved, expectoration much freer, the circulation somewhat mitigated, and she slept soundly at night.

She continued under my care till the 30th of August, during which period she had been regular in using the iodine mixture, in form of vapour, and also the mist. ferri; and at the same time, so much had she improved, that had it not been that her sputa, though much diminished in quantity, still continued purulent, I should have pronounced her cure complete. When she went home, she took a supply of medicines with her; but her husband wrote me, some time afterwards, that she had given up the inhalation, as the use of it distressed her much, and she got considerably worse. Again he wrote about a month ago, for a fresh supply of the iodine mixture, and also for a few bottles of the ferri mixture; and I learned from her sister, a few days since, that she is again much better.

I have only left myself room to say, that from the very favorable issue of a great majority of these cases, my opinion of the influence of the inhalation of iodine and conium is highly favorable, and you are at full liberty to make any use of this letter that you think proper. With many thanks for your disinterested kindness to me, and with every good wish, I am, with the highest respect,

Yours ever,

A. Davidson.

Setham, January 22, 1838."

I indulge the hope that my present communication, and these unreserved details, will procure a large share of increased attention to the subject of inhalation: from many medical friends I have received the most agreeable assurances of their approbation of it. Unfortunately the larger number of cases of this melancholy disease, tuburcular phthisis pulmonalis, admit only of palliative relief; but even in those the inhalation is not only admissible, but very useful in alleviating the symptoms.

A medical gentleman, in the last stage of the disease, came to me, from a considerable distance, in the hope of procuring some relief to his sufferings, which were unusually great and complicated. He was fully aware that he had no probable chance of recovery, but much desired to try the inhalation of iodine and conium. He received such sensible relief from it, that he repeatedly declared he was fully repaid for all the exertions he had made in taking the journey, in the unexpected mitigation of the cough, expectoration, breathing, and general feelings of the chest.

I should too much lengthen this article were I to advance all the arguments which are at my command, derived from experience, in favour of the combined method of treatment which it is my wish to advocate. I say *combined*, for I should regret to be considered as placing my confidence in inhilation alone.

Every one must admit the necessity of seeking for new remedies in phthisis pulmonalis. Is it not true that when once a case is pronounced to be consumptive, the sentence of death is more than half uttered? Urged alike by science and humanity, let us increase our exertions to overcome any particular disease, in proportion to the difficulties by which we are assailed.

As one very leading principle of practice, I apprehend that we should study to overcome the tuburcular diathesis,* and to counteract the fresh formation of tubercles; for it very commonly happens that the relapse of the patient is owing to this cause. This comprehensive view can only be acted upon by using all the means, general and specific, which we think may be best calculated to effect a change in the circulating mass of blood. In addition to the medical treatment, all the juvantia are to be sought for in the general regimen, and all the lœdentia are to be avoided; and without which care, no great disease can be cured.

In the employment of the inhalation, perseverance is necessary, and, in some instances, for many months. The object sought to be obtained is not merely palliative benefit—not merely a temporary impression on the morbid function—but the superseding of the diseased action by a healthy one, and the effecting some organic change.

Many other medicines may be employed usefully in the way of inhalation for tubercular irritation, chronic bronchitis, spasmodic asthma,

* Lately I had an opportunity of seeing the character of hereditary tubercular disease strongly exemplified, in an infant that died in a state of the most extreme emaciation, at the age of four months. The lungs, liver, and spleen, were universally and very closely studded with tubercles; some minute, some large, some hard, others partly or wholly softened. Nor were the intestines free. The mother had died a month before, from the worst complication of pulmonary and intestinal disease I have ever seen; the lungs broken up into cavities, ulcers in the intestines, and tubercles in their coats.

and certain conditions of tracheal disease. These, with the exception of creosote and purified acetic acid, I have noticed in my publication. For the tubercular disease of the lungs, it is in the use of the iodine and conium alone that I place my hopes as a curative agent; I will not say confidence, for that never can be entirely felt in any known treatment of this most fatal of all diseases.

I conclude with expressing my hope that my professional brethren will make fair trial of the plan which I have recommended; and I shall be obliged by receiving communications from them, whether in favour of or against it, for truth is my object. I trust that none will condemn the practice without experience, for this would be prejudice and injustice.

---

6. *Medico-Legal considerations on Monomania; by M. Marc.*—The criminal records of every country afford abundant evidence, that the scaffold in former times has had many victims, who would now be confined in lunatic asylums, or otherwise treated as insane. "It was reserved for *Pinel*," says the author, "to designate the extraordinary position in which, without any sensible abberation of the intellectual faculties, persons are led to acts which, in the eyes of the vulgar, are only explicable on the grounds of extreme depravity. At a later period, his distinguished pupil, *Dr. Esquirol*, established and developed the doctrine of partial delirium, or monomania, which is characterized by a small number of fixed, dominant, and exclusive ideas, and even in one idea alone, to which the insanity is confined, the being, with regard to other objects, perfectly sound." Several examples are given of murders perpetrated by monomaniacs, some of which seemed to be the result of blind and instructive impulse independent of the will, and others on certain processes of reasoning which it is difficult to understand or to develope. Various examples have occurred in this country, in which misplaced sympathy, and the love of the romantic, have elevated to a singular species of dignity, the prepetrators of some of the most henious offences, and thus invested them with an interest which has sometimes had a powerful and unfortunate influence on weak and susceptible minds.

Of this effect the author gives an instance in the case of *Augusta Strohm*, who, when very young, had attended at Dresden the execution of a woman named *Schaefer* for murder. The care with which the culprit was prepared for death, the procession to the scaffold, the execution, produced on Augusta Strohm an impression of which she could never get rid; and from this time she thought that it would be the greatest blessing to end her life in the same way, to have the same preparation for death, and to have an exit equally edifying with that of Schaefer. The idea never quitted her; but her moral principles struggled against it, till the occurrence of another execution for murder, that of a man named *Kaltofen*, which took place at Dresden many years afterwards, when she was about thirty. The deportment of this man before the great number of persons who visited him in prison; the presence of a priest, who never ceased to pray with him; the hypocrisy of the culprit himself; the imposing appearance of the military escort which conducted him to the scaffold; the immense crowd of spectators, and the sentiment of compassion which, in spite of the enormity of the offence, depicted itself on the countenances of many of them; the calm aspect of the condemned person; his address to the people; the approach of the priest to soften the last moments of his life, together with the promptitude and apparent mildness of the kind of death which he suffered, changed into a fixed resolution the perverted feelings of Augusta Strohm.

Six weeks afterwards, she invited a fine young woman, a very particular friend, to take coffee, and availed herself of an accidental slumber, to attack her with a hatchet. This not sufficing for her destruction, the consummation of the deed was perpetrated, after a faint struggle, by stabs on the breast with a knife. Strohm then cleansed and abjusted the room, placed the body on the bed, and lay down beside it, with the intention of passing the night. But when daylight began to disappear she was seized with a tremor and oppression, and determined immediately to deliver herself up to the hands of justice, which she only meant to do the following morning. She then drest herself with care, took with her a prayer book, and also money and linen, which she knew would be necessary during her imprisonment, and forthwith presented herself to an officer of police, as the murderer of her friend, whose body she represented would be found in her appartment. We are left to infer the close of the catastrophe.—*Edinburgh Medical and Surgical Journal.*

